# Supplementary material for: Antioxidant and Cytoprotective Capacities of Various Wheat (Triticum aestivum L.) Cultivars in Korea
Source: Foods. 2022 Aug 5;11(15):2338. doi: 10.3390/foods11152338 (PMC9368500; doi:10.3390/foods11152338)
Supplement: Supplementary file 1 [file foods-11-02338-s001.zip › foods-1826480-supplementary.pdf]

Supplementary Materials

Antioxidant and Cytoprotective Capacities of Various Wheat (*Triticum aestivum* L.) Cultivars in Korea

Huijin Heo [1], Hana Lee [1], Jinhee Park [2], Kyeong-Hoon Kim [2], Heon-Sang Jeong [1] and Junsoo Lee [1,*]

[1] Department of Food Science and Biotechnology, Chungbuk National University, Cheongju 28644, Korea; pltreasure11@gmail.com (H.H.); dlgksk0514@naver.com (H.L.); hsjeong@chungbuk.ac.kr (H.-S.J.)

[2] Wheat Research Team, National Institute of Crop Science, Rural Development Administration, Wanju 55365, Korea; pjh237@korea.kr (J.P.); k2h0331@korea.kr (K.-H.K.)

* Correspondence: junsoo@chungbuk.ac.kr; Tel.: +82-43-261-2566

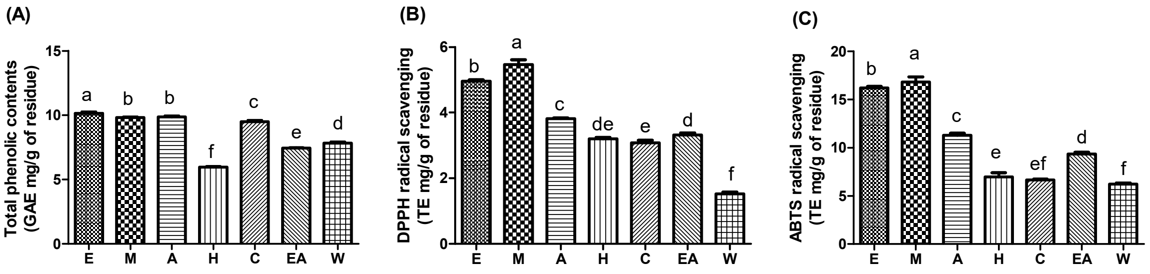

Figures S1. Total phenolic contents (A), DPPH radical scavenging activity (B), and ABTS radical scavenging activity (C) in Keumkang wheat extract by various extraction solvents. All values are the means ± standard deviation (n=3). Different letters (a-f) in each figure indicate a significant difference by Duncan's multiple range test ($P < 0.05$). E, ethanol; M, methanol; A, acetone; H, hexane; C, chloroform; EA, ethyl acetate; W, water.

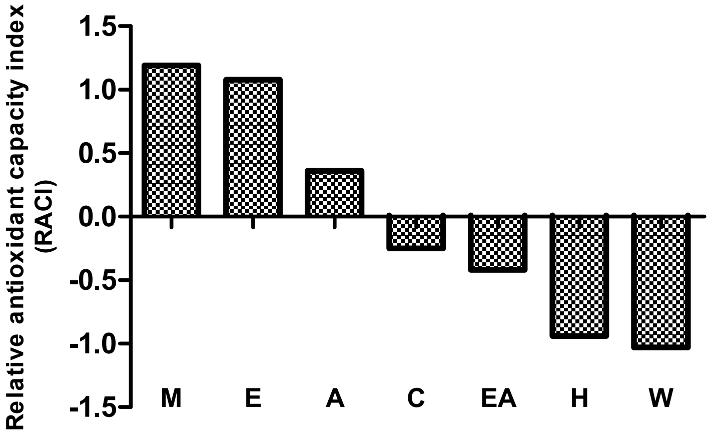

Figures S2. Relative antioxidant capacity index (RACI) of Keumkang wheat extract by various extraction solvents. M, methanol; E, ethanol; A, acetone; C, chloroform; H, hexane; EA, ethyl acetate; W, water.

Table S1. Antioxidant and cytoprotective capacities of 41 wheat cultivars.

No.	Cultivars	RACI[1]	TPC[2] GAE[3] mg/g residue	DPPH TE[4] mg/g residue	ABTS TE mg/g residue	Cytoprotective capacities[5] (%)		
						Hepatocytes	Myoblasts	Endothelial cells
1	Ol	-1.38	9.62	3.48	38.79	27.46	59.41	0.00
2	Geuru	-0.13	10.55	3.67	43.41	22.37	74.61	40.20
3	Dahong	0.02	11.26	3.36	47.17	42.83	57.48	41.96
4	Chungkye	-0.43	10.64	3.49	42.87	43.85	79.48	40.59
5	Eunpa	0.80	11.66	3.66	46.63	43.85	51.85	4.71
6	Tapdong	-1.96	10.05	3.38	27.73	11.69	78.01	0.00
7	Namhae	0.40	10.95	3.81	43.68	103.26	76.93	0.00
8	Uri	0.43	11.12	3.59	48.41	47.86	55.09	0.00
9	Olgeuru	-0.60	10.37	3.41	45.02	15.66	0.00	58.91
10	Alchan	0.17	10.91	3.65	44.86	74.39	3.12	47.40
11	Gobun	-0.88	10.75	3.17	43.63	82.37	0.00	102.48
12	Keumkang	-0.37	10.57	3.51	43.89	77.25	29.68	44.43
13	Seodun	-0.80	11.09	3.43	33.96	100.96	15.94	54.58
14	Saeol	0.18	10.90	3.82	40.46	30.25	0.00	37.75
15	Jinpoom	0.09	10.85	3.50	48.67	91.71	11.20	65.59
16	Milsung	0.15	10.71	3.89	39.76	95.75	0.00	68.81
17	Joeun	-0.47	10.61	3.44	44.00	72.81	45.98	74.64
18	Anbaek	0.42	11.05	3.41	54.42	88.26	32.90	51.18
19	Jopoom	0.50	10.78	3.84	46.04	116.71	84.91	37.14
20	Shinmichal	0.39	11.60	3.71	39.28	83.29	92.96	7.07
21	Jonong	0.16	11.01	3.53	47.28	77.89	104.89	0.09
22	Jokyung	-0.36	10.89	3.24	48.46	58.77	112.36	8.97
23	Yeonbaek	-1.06	9.92	3.44	41.85	115.26	90.66	0.00
24	Shinmichal 1	0.39	10.78	3.81	45.29	138.29	101.87	9.87
25	Dabun	1.04	11.44	3.74	50.61	112.50	51.04	85.44
26	Baekjoong	-0.22	11.24	3.51	39.12	50.13	31.82	19.19
27	Jeokjoong	-0.93	10.18	3.29	45.51	32.93	71.48	2.82
28	Sukang	-0.74	10.22	3.44	43.84	19.23	67.56	37.70
29	Hanbaek	-0.18	10.61	3.55	45.51	66.45	73.81	0.00
30	Suan	0.59	10.89	3.73	49.64	91.47	48.59	0.00
31	Dajoong	1.71	12.23	3.59	57.16	77.67	106.49	0.00
32	Goso	0.67	11.57	3.59	47.55	33.67	118.97	10.27
33	Joah	-0.02	11.24	3.68	37.45	40.06	51.05	98.52
34	Hojoong	0.72	11.16	3.68	50.28	58.01	81.93	85.35
35	Baekchal	0.06	10.35	3.81	44.54	78.12	52.88	43.95
36	Jojoong	0.77	11.30	3.65	50.34	68.10	44.57	118.41
37	Baekkang	-0.68	10.39	3.22	49.10	85.49	73.73	42.20
38	Seaekeumkang	0.62	10.75	3.79	49.80	86.57	69.51	45.03
39	Taejoong	0.77	11.07	3.79	48.57	33.06	78.05	75.94
40	Johan	-0.30	11.14	3.40	42.18	16.78	64.58	64.38
41	Hwanggeum	0.46	11.10	3.57	49.69	9.75	66.46	110.08

[1] Relative antioxidant capacity index

[2] Total phenolic contents.

[3] Gallic acid equivalents.

[4] Trolox equivalents.

[5] Cytoprotective activities calculated by equation (1) and (2) in main article.
